# Acute Glanders Communicated by the Bite of a Horse Labouring under Chronic Disease

**Published:** 1844-11-16

**Authors:** 


ACUTE GLANDERS COMMUNICATED BY THE BITE OF A HORSE LABOURING UNDER CHRONIC DISEASE.

An individual, whilst administering medicine to his diseased horse, was accidentally bitten on the cheek. Two days subsequently, symptoms of acute glanders manifested themselves. The patient died after an illness of thirteen days, having presented during life the usual symptoms of this disease, and in addition there appeared one not heretofore described—opacity of the cornea.

"It was further remarked, as the horse had been five months ill, and as the case was one of acute glanders, communicated by a bite from an animal then chronically diseased, that the distinction which some persons had attempted to establish between acute and chronic glanders was not warranted."—*Veterinarian.*

